# Biological Potential of the Main Component, Thymoquinone, of *Nigella sativa* in Pulp Therapy—In Vitro Study

**DOI:** 10.3390/life12091434

**Published:** 2022-09-15

**Authors:** Rana A. Alamoudi, Soha A. Alamoudi, Ruaa A. Alamoudi

**Affiliations:** 1 Pediatric Dentistry Department, Faculty of Dentistry, King Abdulaziz University, Jeddah 21589, Saudi Arabia; 2 Biological Sciences Department, College of Science and Arts, King Abdulaziz University, Rabigh 21911, Saudi Arabia; 3 Endodontic Department, Faculty of Dentistry, King Abdulaziz University, Jeddah 21589, Saudi Arabia

**Keywords:** *Nigella sativa*, antimicrobial activity, root canal, microorganisms, thymoquinone

## Abstract

This work is designed to assess the antimicrobial efficacy, chelating efficacy, and dissolving capability of the bioactive agent of the *Nigella sativa* plant (thymoquinone). Four freeze-dried microorganisms were studied. Each species was treated with either 6% sodium Hypochlorite, thymoquinone, or sterile water. The zone of inhibition was measured. Thirty extracted human premolar teeth were utilized to evaluate the smear layer removal. Root canals were mechanically instrumented and then irrigated with either 6% sodium Hypochlorite, 17% ethylenediaminetetraacetic acid, or thymoquinone for 1 min and scanned under the scanning electron microscopic to evaluate the cleanliness of the root canal and the remainder of the smear layer. To evaluate the tissue dissolving effect, Bovine Pulp Tissue was utilized. Randomly treated samples included: 6% sodium Hypochlorite, thymoquinone, or isotonic saline for 30 min. The remaining pulp tissue was weighed. Thymoquinone reported the highest inhibition of microbial multiplication compared to other irrigants (*p* < 0.001). Thymoquinone solution had an excellent antibacterial effect on endodontic pathogen and did not affect the inorganic and organic tissue inside the root canal. Meanwhile, it reported weak chelating and dissolving effects. Tissue dissolution was statistically significant with sodium Hypochlorite solution compared to other groups (*p* < 0.001).

## 1. Introduction

Endodontic infections are polymicrobial infections that contain mainly anaerobic bacteria and facultative species [1]. Root canal treatment helps to eradicate microbes and prevent recontamination after chemo-mechanical preparation [2]. During root canal treatment, different irrigants are required to be used. An ideal endodontic irrigant possesses an antibacterial effect, tissue dissolving ability, and smear layer removal. It is essential that endodontic irrigants be non-poisonous for the adjacent cells and do not damage the tooth construct [3]. Sodium hypochlorite (NaOCl) is the most recommended irrigant. The NaOCl concentrations are in the range of 0.5–6% and have various killing efficacy. Different studies reported its strong antibacterial properties [4] and tissue dissolving capability [5]. However, NaOCl is cytotoxic to the periapical tissue [6] and cannot remove inorganic particles from the smear layer [7].

Another irritant, 17% Ethylenediaminetetraacetic acid (EDTA), is usually used in conjunction with NaOCl. This irrigant is characterized by its potential to melt the inorganic section of the dentine and smear layer. Recently, Qianet al. [8] advocated that using NaOCl after EDTA can cause an extensive erosion to the root canal dentinal wall and subsequently weaken the tooth. Natural materials were recently introduced as promising materials in different aspects of dental remedies, such as caries control and treatment of gingivitis. An old aromatic black cumin plant known as *Nigella sativa* (*N. sativa*) is widely planted, especially in the Middle East and Southeast Asia. The seeds of this herb have been utilized to stimulate health for centuries. *N. sativa* seeds consist of 22.7% proteins, 41% fixed oil, 1.6% yellowish volatile oil, amino acids, minerals, and vitamins. Different studies reported its biological properties, including antibacterial, antifungal, and anti-inflammatory [9,10]. Thymoquinone is the bioactive agent in the *N. sativa* plant responsible for its antibacterial property, and its activity can enhance antibiotic actions [11]. Previous research reported the impact of thymoquinone on oral bacteria [12]. On the contrary, thymoquinone has some toxic effects, where Zhang et al. [13] revealed that TQ treatment triggers the apoptotic pathway induced by p-p38 MAPK and simultaneously leads to the upregulation of Erk phosphorylation. Phosphorylated Erk or JNK has been reported to inhibit apoptosis and produce drug resistance in tumor cells by maintaining the BCL-2 and Bad binding. No one showed its effect on root canal bacteria. In addition, a recent randomized clinical trial showed the effect of thymoquinone in eliminating renal stones. The n-butanol portion and n-butanol stage remnant can effectively dissolve calcium oxalate deposits from kidney stones [14,15,16]. Thus, this work aims to evaluate the antimicrobial, chelating, and dissolving capability of the *N. sativa* plant (thymoquinone).

## 2. Materials and Methods

The procedures of this work were accepted by King Abdulaziz University’s ethical committee (# 253-07-21). Thymoquinone was attained from the Sigma Company (St. Louis, MO, USA), which was prepared by dissolving dimethyl sulfoxide (DMSO) and cleaned with an inert gas. The solubility of thymoquinone in DMSO is nearly 14 mg/mL.

### 2.1. Evaluation of the Antibacterial Effect

#### 2.1.1. Preparation of Microbial Suspension

Four freeze-dried bacteria strains: *Streptococcus sanguis* (ATCC 10556), *Enterococcus faecalis* (ATCC 29212), *Prevotella intermedia* (ATCC 25611), and *Porphyromonas gingivalis* (ATCC 33277) were chosen in this study according to their higher percentage presence in root canal infection. The bacterial strains were obtained from Trust Medical Supplies, Jeddah, Saudi Arabia. Two types of media were selected based on bacterial types on this study. Brain Heart Infusion (BHI) agar (ATCC medium 44) was the media chosen for *Streptococcus sanguis* and *Enterococcus faecalis*. Tryptic Soy (TS) agar (ATCC medium 2722) was the media selected for *Prevotella intermedia* and *Porphyromonas gingivalis*. For media preparation, BHI and TS agars were prepared and autoclaved at 121 °C for 20 min.

For strain suspension preparation, stock cultures of each strain were cultured individually in an agar plate and then incubated overnight at 37 °C to allow us to extract the bacteria stain. A single colony from each of the aerobic bacteria (*Streptococcus sanguis* and *Enterococcus faecalis*) were isolated and mixed individually in 1 mL of BHI broth. Additionally, another single colony from each of the anaerobic bacteria (*Prevotella intermedia* and *Porphyromonas gingivalis*) were extracted and mixed separately in 1 mL of TS broth. The suspensions were incubated at 37 °C at 250 rpm for 24 h to be ready for antimicrobial activity screening tests.

#### 2.1.2. Screening for Antimicrobial Activity

New BHI and TS agar plates were inoculated with 100 μL aliquot of isolate suspension (Optical dentistry 600 nm “OD600”). For each bacterium, plates were allocated into three groups (n = 10). The first group received 6-mm sterile paper disks soaked with 6% NaOCl solution (Ultra Clorox^®^ Germicidal Bleach; Clorox^®^, Oakland, CA, USA) and functioned as the positive control. The second group received 6-mm thymoquinone-filled discs. The third group received sterile water discs (Braune Medical Inc., Irvine, CA, USA) and acted as a negative control. All plates were incubated at 37 °C, and the obligate anaerobes bacteria were incubated in a special anaerobic chamber. All plates were incubated for 48–72 h. Zones of inhibition were determined using a digital caliper (No. 721A, Electronic Digital Calliper; LS Starrett Co., Athol, MA, USA).

### 2.2. The Removal of Smear Layer

#### 2.2.1. The Root Canal Preparation

Thirty extracted individual premolar teeth were utilized. Teeth were stored in a thymol disinfectant agent for 48 h and then kept in distilled water till use. Two periapical radiographs were obtained for every tooth in two directions, buccolingual and mesiodistal. Teeth were chosen if they were sound and had no caries or cracks, if there was no previous root canal treatment, no external or internal resorption, no calcification, and the mature apex with root curvature was less than 10 degrees.

All teeth were decoronated at the cementoenamel junction via diamond bur. The roots were sectioned to establish a 12-mm length; then, the working length was calculated by deducting 1 mm from the entire root length. A size 10-K file (Dentsply Maillefer, Ballaigues, Switzerland) was employed to confirm patency. Root canals were equipped with ProTaper Universal instruments (Dentsply Tulsa Dental, Tulsa, OK, USA) up to size F3 (size 35, 0.06 taper). Specimens were rinsed with 2 mL of 6% NaOCl (Ultra Clorox^®^ Germicidal Bleach; Clorox^®^, Oakland, CA, USA) between each instrument via a 27-gauge Max-i-Probe needle (Dentsply Maillefer, Ballaigues, Switzerland).

Specimens were distributed in a random method into three groups (n = 10). Group 1 was rinsed with 5 mL of 17% EDTA (Sigma-Aldrich Corp., St Louis, MO, USA) for one minute to act as a positive control. Group 2 was rinsed with 5 mL thymoquinone extract and left in the canal for one minute, and Group 3 was rinsed with 5 mL of normal saline for one minute to function as a negative control group. All specimens were washed with 5 mL distilled water to eliminate any residual solutions and then blotted with sterilized filter paper points.

#### 2.2.2. Scanning Electron Microscopic Analysis

Markings were created at 2, 4, and 6 mm from the tip of each root horizontally cut with a diamond disc using water as a coolant. A cone of Gutta-percha was put into the root canal to cover the canal walls and reduce debris from the sectioning. The teeth were coated with gold-palladium sputter (Agar Sputter Coater B7340; Agar Scientific Ltd., Stanton, UK) to prepare a scanning electron microscope (JEOL Ltd., Tokyo, Japan) at 1000× magnification, and photomicrographs were taken and evaluated. Two independent examiners were trained to determine the presence of a smear layer according to Torabinejad’s et al. [17] description, where a score of zero has no smear layer on the surface of the root canal, and all dentinal tubules are clean and open, and score one has a moderate smear layer on the surface of the root canal. However, there is debris in the dentinal tubules, and a thick smearing layer covers the root canal surface and dentinal tubules in score two. The Kappa (k) statistic was used to assess inter- and intra-observer agreement using the following interpretation paradigm: poor (0.20), fair (0.21–0.40), moderate (0.41–0.60), good (0.61–0.80), and excellent (0.81–1.00) [18].

### 2.3. Evaluation of the Tissue Dissolving Effect

#### 2.3.1. Bovine Pulp Tissue Preparation

This study utilized thirty freshly extracted bovine mandibular incisors. This investigation was not categorized as an animal study since our work will have no decision on the slaughtering process of the animal. The animals were slaughtered for commercial uses, and the teeth were extracted from bovine jaws and stored at −20 °C until needed. Then, at the time of the work, teeth were kept at room temperature until defrosted. All crowns were sectioned at the cementoenamel junction via a diamond bur. Pulp tissues were carefully extirpated using hemostatic forceps, rinsed with water to eliminate debris and clotted blood, and then dried. Thirty pulp tissues were collected and divided into three groups. The primary weight of every group was evaluated using a precision balance Mettler Toledo ME204 (Columbus, OH, USA) in an airtight container. Each weighing 30 ± 5 mg was made with a #12 surgical blade.

#### 2.3.2. Dissolution Experiment

Pulp tissue samples were divided into three groups of ten (n = 10). The last weighted bovine pulp pieces were placed in Eppendorf test tubes and soaked in one of the solutions: Group 1: 5 mL of 6% NaOCl solution (Ultra Clorox^®^ Germicidal Bleach; Clorox^®^, Oakland, CA, USA). Group 2: 5 mL of thymoquinone. Group 3: 5 mL of isotonic saline solution (0.9% NaCl) (IEUlagay Drug Industry, Istanbul, Turkey). The sample was continuously weighted every five minutes for 30 min.

### 2.4. Statistical Analysis

For growth inhibition zones and the presence of a smear layer, a Kruskal–Wallis test was used to define significant differences among the different irrigations since the Bartlett test showed that the group variances were not homogenous and not normally distributed. Group pairs were assessed using the Mann–Whitney U test for statistical significance. In the tissue dissolving test, the nonparametric Friedman test (*p* < 0.05) was utilized to determine the intragroup differences between various durations of immersion, and the Kruskal–Wallis test (*p* < 0.05) was applied to define intergroup differences at the same time. Significance was determined at *p* < 0.05. Statistical analysis was adopted via SPSS Window software (Version 16.0; SPSS, Inc., Chicago, IL, USA).

## 3. Results

**Figure 1:**
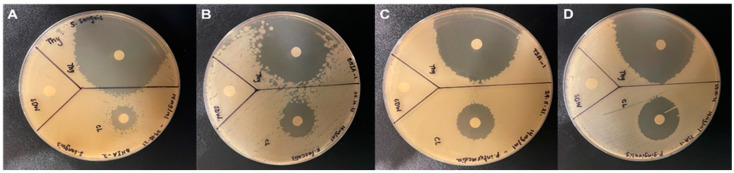
Zone of inhibition for three irrigants: NaOCl, Thymoquinone, and Water against (**A**) *Streptococcus sanguis*; (**B**) *Enterococcus faecalis*; (**C**) *Prevotella intermedia*; (**D**) *Porphyromonas gingivalis*.

The inter-and intra-examiner reproducibility varied from good to excellent. The Kappa values for inter-examiners were about 0.80, while the Kappa values for intra-examiners were 0.84. The Mann–Whitney test revealed the differences between different irrigants at room temperature; Thymoquinone reported the highest inhibition of bacterial multiplication of all other irrigants evaluated (*p* < 0.001), as shown in Figure 1. The Kruskal–Wallis test reported no significant difference in the antibacterial impact of each solution on the four types of bacteria (*p* = 1.00), as reported in Table 1. However, for each bacterium, there is a significant difference among the various irrigants (*p* < 0.001).

Concerning the tissue dissolving test, the mean weight, the standard deviation of pulp tissues, and the percentage difference between the initial weight and the weight post immersion over time were reported for each solution in Table 2. Tissue dissolution was statistically significant with NaOCl solution and the immersion time (*p* < 0.001). Water and thymoquinone solutions did not change the weight of fragments among times (*p* > 0.001).

**Figure 2:**
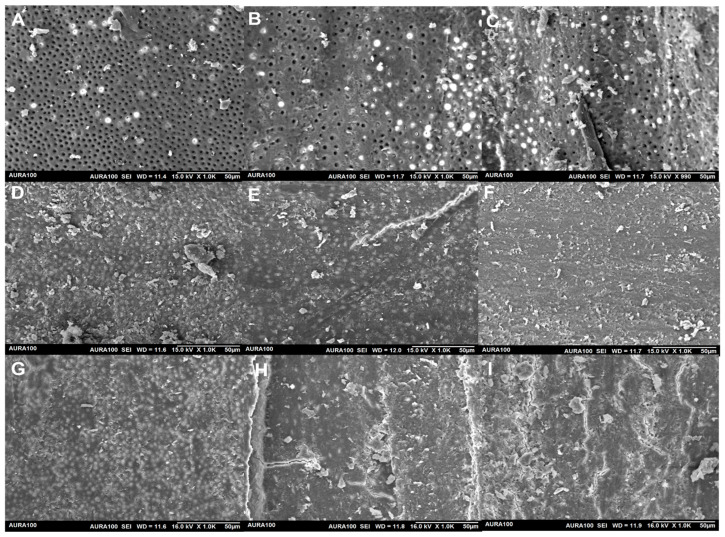
Illustrative SEM samples of each group (original magnification 1000×): incorporation of EDTA at coronal third level (**A**); inclusion of EDTA at mid-root level (**B**); addition of EDTA at the apical third level (**C**); Thymoquinone irrigated-coronal third level (**D**); mid-root level irrigated by Thymoquinone (**E**); Thymoquinone irrigation at the apical third level (**F**); water irrigation at the coronal third level (**G**); mid-root level irrigated water (**H**); water irrigation at the apical third level (**I**).

The data of the SEM are summarized in Table 3. In the coronal part, the EDTA group presented with a dentin surface devoid of a smear layer with a statistically significant difference in contrast to thymoquinone and water solutions (*p* < 0.001). In the mid-root and apical third sections, the dentin surface of the canal was cleaner in the EDTA group contrasted to the thymoquinone and water groups (*p* < 0.029), but the difference was not significant. Both thymoquinone and water revealed a dentin surface with a smear layer and debris with no statistically significant difference between both solutions (*p* = 1.00) (Figure 2).

## 4. Discussion

*Nigella sativa* is a promising herb with many therapeutic indications and has not been the subject of wide-ranging research recently. Many researchers have attributed the benefits of *N. sativa* to thymoquinone, the main bioactive ingredient of this oil. Thymoquinone is a common nonpolar material in the extracts of *N. sativa* seeds [19,20,21,22,23,24]; it comprises 18.4% to 24% of the *N. sativa* volatile oil [25]. Thymoquinone is chemically 2-methyl-5-isopropyl-1, 4-benzoquinone [26]. Thymoquinone has osteogenic, antibacterial, anti-inflammatory, antioxidant, and analgesic impacts while negatively impacting normal cells [27]. Thus, this work was designed to assess the different properties of thymoquinone material important in pulp therapy.

Thymoquinone was tested for its antimicrobial effect against many bacterial, fungal, and parasitic organisms [9,27,28,29]. Our studies evaluated the effect of the thymoquinone as an endodontic irrigant used to eradicate different species of endodontic pathogens. The strains of bacteria were carefully chosen for this as *Streptococcus sanguis*, *Enterococcus faecalis*, *Prevotella intermedia*, and *Porphyromonas gingivalis* are portions of the endodontic microbal flora and were presented in either an inflamed vital pulp or a necrotic infected one [1,30,31].

The present study revealed a potent antibacterial effect of the thymoquinone with a clear zone of inhibition from 60–75 mm. This result is consistent with Khattab and Omar’s, which found that thymoquinone significantly decreased the infected root canals’ microbial flora [32]. The mechanism of action of thymoquinone relies on the capability to infuse the microbial membrane and destroy its cell. Gram-negative bacteria have an excellent permeability resistance because of the outer structure of the cell membrane. Therefore, thymoquinone has more pronounced antimicrobial impact on Gram-positive than Gram-negative bacteria. The data support previous studies which advocated an excellent effect of thymoquinone versus Gram-positive and Gram-negative bacteria [33,34,35,36,37].

Efflux is an essential process in bacterial resistance [38,39]. The efflux pumps (EPs) are proteins in the bacterial membranes that allow antimicrobial substances’ extrusion from the cell. These pumps transport antimicrobial agents via the bacterial envelope and minimize intracellular accumulation [40]. Since thymoquinone is hydrophobic, it may be able to penetrate the outer membrane of bacteria to affect membrane integrity, which in turn can show co-aggregation of these species. It was recorded that thymoquinone provides a rich source of efflux pumps inhibitors (EPIs) [41,42,43,44,45,46]. Thus, this solution is urgently needed with new modes of action in cases of antimicrobial resistance.

Another promising result of this work was that the antimicrobial impact of the thymoquinone is concentration-dependent. According to other studies, the lowest level of thymoquinone to stop *Enterococcus faecalis* is 256 μg/mL [35]. The current study reported that all strains showed a zone of inhibition at 7 mg/mL concentration. Meanwhile, the thymoquinone concentration was doubled to 14 mg/mL, showing a higher inhibition zone. Thus, the effectiveness of thymoquinone as an antibacterial agent depends on its level.

This study also assesses the effect of thymoquinone in removing inorganic material. Several studies reported that thymoquinone disrupts calcium oxalate crystals formed on kidney calculi in rats [47,48]. Thymoquinone could inhibit stone formation by decreasing crystal deposition and increasing glutathione peroxidase superoxide dismutase [49,50]. However, the present study did not show any chelating properties on the smear layer. The smear layer consists of microcrystalline and organic particle debris (45% of dentin composed of the mineral hydroxyapatite, 33% is an organic material, and 22% is water). The result may be explained by hydroxyapatite crystal in the smear layer consisting of calcium phosphate (Ca_3_(PO_4_)_2_), while the kidney calculi are calcium oxalate (CaC_2_O_4_). These two compositions are associated with different metabolic milieus and respond differently to different chemicals.

The present study also assessed the effect thymoquinone has on dissolving organic tissue. The results indicate that this material does not have any dissolving effect. Nevertheless, an in vitro study advocated that thymoquinone elevates the total antioxidant impact and migration potential of mesenchymal cells [49]. Researchers studied the impact of thymoquinone on cell proliferation and noticed that it could stimulate the formation of fibroblasts & collagen production throughout wound repair in rabbits [51]. Others found that thymoquinone could stimulate dental pulp mesenchymal cells’ osteogenic differentiation [52,53].

Moreover, thymoquinone exerts an anti-inflammatory effect on some inflammatory mediators such as leukotriene [54,55]. The potent antioxidant effect of thymoquinone is due to different free radicals. It can also depress the cyclooxygenase and lipoxygenase pathways of arachidonic acid metabolism. Tekeoglu et al. [56] found that the levels of TNF-a and IL-1b in the thymoquinone-treated group were markedly lower than in other groups. Thymoquinone showed a slight inflammatory reaction and tissue disorganization compared to the calcium hydroxide group, which showed a more inflammatory response and PMNs infiltration and slight tissue disorganization [57].

## 5. Conclusions

The *N. sativa* plant (thymoquinone) exhibited an excellent antibacterial effect on endodontic pathogens. However, it reported weak chelating and dissolving effects. It is worth mentioning that thymoquinone has diverse pharmacological actions and thus becomes an attractive material for developing different products since it has low toxicity toward human cells.

## Figures and Tables

**Table 1:** Growth inhibition diameters (mm) of irrigants versus four types of bacteria. The results recorded in means and standard deviation (SD).

Treatments	*Streptococcus sanguis*	*Enterococcus faecalis*	*Prevotella intermedia*	*Porphyromonas gingivalis*
**Water**	0	0	0	0
**NaOCl**	43 ± 5.42	46 ± 3.38	32 ± 4.21	37 ± 2.06
**Thymoquinone**	72 ± 4.08	66 ± 4.38	62 ± 3.50	60 ± 4.08

**Table 2:** Mean (X) and standard deviation (SD) in mg of the weights of bovine pulp tissue fragments prior and post different durations of immersion in the irrigators and the decrease in weight of the fragments in percentage.

Treatments	IW * X ± SD	Weight Post 5 min. of Immersion		Weight Post 10 min. of Immersion		Weight Post 15 min. of Immersion		Weight Post 20 min. of Immersion	
		X ± SD	FWR * (%)	X ± SD	FWR (%)	X ± SD	FWR (%)	X ± SD	FWR (%)
**Water**	28.24 ± 1.08	25.42 ± 2.81	10	25.37 ± 2.15	10.2	23.06 ± 3.22	18	20.77 ± 1.67	26.5
**NaOCl**	29.89 ± 1.95	9.48 ± 0.74	68.3	0.30 ± 0.36	99	0	100	0	100
**Thymoquinone**	33.80 ± 1.62	32.16 ± 3.32	4.9	31.71 ± 1.28	6.2	26.36 ± 0.93	22	24.25 ± 1.85	28.3

* FWR, Fragment weight reduction (%), IW, Initial weight.

**Table 3:** Quantity of samples and % concerning the smear layer scores in the root thirds of all trial groups.

Treatments		No Smear Layer	Smear Layer in Dentinal Tubules, Clear Dentinal Surface	Smear Layer in Dentinal Tubules and Surface
**Water**	Coronal third	0 (0%)	0 (0%)	10 (100%)
	Middle third	0 (0%)	0 (0%)	10 (100%)
	Apical third	0 (0%)	0 (0%)	10 (100%)
**EDTA**	Coronal third	4 (40%)	6 (60%)	0 (0%)
	Middle third	4 (40%)	5 (50%)	1 (10%)
	Apical third	2 (20%)	4 (40%)	4 (40%)
**Thymoquinone**	Coronal third	0 (0%)	0 (0%)	10 (100%)
	Middle third	0 (0%)	0 (0%)	10 (100%)
	Apical third	0 (0%)	0 (0%)	10 (100%)

## Data Availability

The data presented in this study are available on request from the corresponding author.
